# Antecedents and Consequences of Online Healthcare Community Usage: A Grounded Theory Approach

**DOI:** 10.3390/healthcare10091749

**Published:** 2022-09-12

**Authors:** Zhanyou Wang, Xin Zhang, Dongmei Han, Liang Ma

**Affiliations:** 1 Management Science and Engineering, Shandong University of Finance and Economics, Jinan 250014, China; 2 School of Labor Relations, Shandong Management University, Jinan 250014, China; 3 School of Information Engineering, Shandong Management University, Jinan 250014, China

**Keywords:** online healthcare community, doctor–patient relationship, doctor–patient communities, patient satisfaction, influence factors, grounded theory

## Abstract

The online healthcare community has grown rapidly in recent years. However, the antecedents and consequences of the use of online healthcare community platforms have not been systematized. Using grounded theory, this study collects first-hand data on the use of online healthcare communities through in-depth interviews and analyzes the interview data using a three-level coding approach including open coding, axial coding, and selective coding. The results showed the following. (1) Doctors, hospitals, and the online platforms themselves, along with the impact of healthcare environmental factors, affect patients using the online healthcare community. (2) The use of an online healthcare platform affects patient-related factors, such as emotional dependence and patient satisfaction, as well as factors related to doctor–patient interactions, such as the perception of healthcare quality and the doctor–patient relationship, through mediating factors, including doctor–patient communication, treatment processes, and healthcare costs. (3) Improving patients’ healthcare experience and the doctor–patient relationship may feed back into operation quality and the operating environment of the online healthcare community, thus promoting the promotion and use of an online healthcare community platform.

## 1. Introduction

Previous research has explored and made outstanding contributions to the understanding of online healthcare community platforms and the relationship between doctors and patients [1,2,3]. However, it is worth noting that most previous studies on the relationship between the use of online healthcare communities and doctor–patient interactions have been conducted from the perspective of single patient and doctor, and their conclusions have not been entirely consistent [4,5]. The reasons for this are as follows: the dimension, intensity, and platform characteristics of the use of online healthcare communities affect the mechanisms of the doctor–patient relationship. In existing results, the influence, a comprehensive model, and the boundary conditions of different dimensions the use of online healthcare community on the doctor–patient relationship remain unclear [6]. It is worth mentioning that the existing results are largely derived from quantitative analysis, which is insufficient for describing relatively clear mechanisms of the influence that the use of online healthcare communities has on the doctor–patient relationship [7,8]. However, the unclear mechanisms of formation lead to a lack of guidelines in the management of online healthcare community platforms, making it difficult to develop targeted management strategies to improve performance [9,10]. Therefore, this paper focuses on the following research questions. First, what are the factors and dimensions that affect the use of an online healthcare community platform? Second, how do these online healthcare communities affect the doctor–patient relationships, and what are the mechanisms of this action and its boundary conditions?

This paper takes a grounded theory approach to conduct exploratory research. On the one hand, this approach sorts out and summarizes the key factors and dimensions affecting the use of the online healthcare community. Another aspect of this work is in exploring the mechanisms and boundary conditions used by online healthcare communities to influence the doctor–patient relationship. On this basis, the mechanism of the influence of the use of the online healthcare community on the doctor–patient relationship is constructed. The specific process is divided into eight steps: that define the phenomenon of the use of online healthcare communities, condensing the problems of online healthcare communities, selecting research objects, carrying out theoretical sampling, collecting data, carrying out three-level coding (open coding, axial coding, and selective coding), comparing and reviewing citations in the literature, and forming a theoretical model. To clarify the key elements and theoretical model of the mechanism of the influence process of the use of online healthcare community on the doctor–patient relationship, it is necessary to acquire first-hand interview data from actual users of online healthcare community through interviews and then analyze the data obtained to create a theoretical model. Semi-structured interviews are conducted with 18 users of online healthcare communities from different regions. Then, based using grounded theory, this paper summarizes the use of different purposes and dimensions in the online healthcare community, exploring process mechanisms and the boundary conditions that affect the doctor–patient relationship. Finally, a conceptual model is constructed, and then a theoretical model is formed through a review and comparison with the literature.

## 2. Research Design

### 2.1. Research Method

Grounded theory, a typical form of qualitative research, was first proposed in 1967 by Glaser and Strauss [11]. It has seen use in various fields of the social sciences [12]. The core of this approach is in the extraction and summary of relevant concepts and categories from actual enterprise data for specific research problems, and finally, raising the phenomena to a theoretical level. The specific research steps of grounded theory are as follows. (1) Asking questions. In this stage, first, the phenomena to be studied should be identified, and then a literature review and field observations can be carried out to better define the phenomena, and then the research problems should be clarified. (2) Collecting data. In this stage, theoretical sampling, collection of objective and subjective data collection (in in-depth interviews, etc.), collation of research data and recording, with some of the samples being reserved for theoretical saturation tests. (3) Analyzing the data. This centers on a three-level coding of grounded theory, namely, open coding, axial coding, and selective coding. (4) Constructing a conceptual model. The main research steps at this stage include obtaining a theoretical model using three-level coding analysis, testing the saturation of the theoretical model, and establishing the theoretical model in the context of this study. In these research steps, the use of three levels of data coding is a crucial step in the application of grounded theory.

**Figure 1:**
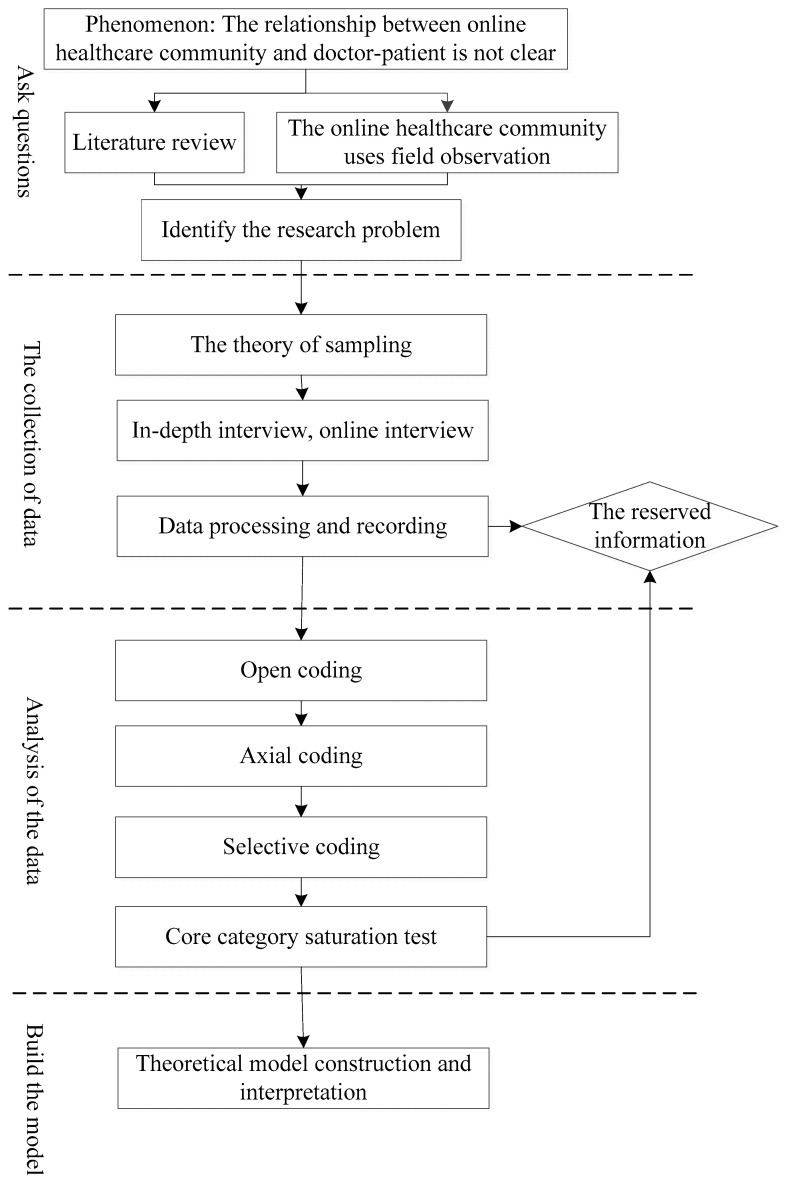
This paper studies the process of grounded theory.

Open coding is used to analyze and organize the data collected by means of conceptualization and categorization to reflect the connotations of the collected data [13]. Axial coding is the process of. using a certain program or model, in-depth excavation of the relationship between categories and the processes of influence, as well as the formation of primary and secondary categories. The most widely used model for axial coding is Strauss and Corbin’s canonical model [14]. Selective coding is the process of selecting core categories based on open coding and axial coding [15]. This paper investigates grounded theory to explore the formation mechanism of the influence of the use of online healthcare community on the doctor–patient relationship, and the process of studying grounded theory is shown in Figure 1 below.

### 2.2. Research Objects

The interview subjects in this study are users of online healthcare communities, including Good Doctor Online, Seeking Medicine, DingxiangDoctor, Jingyitong, DingxiangGarden, and others. These users check healthcare information and patient evaluations, create their own accounts, seek consultations, evaluate doctors, help spread healthcare knowledge, and monitor the COVID-19 pandemic online. To prevent deviation from the research theme, each interview was conducted according to a standard interview outline designed in the early stages of the work.

### 2.3. Data Collection

**Figure 2:**
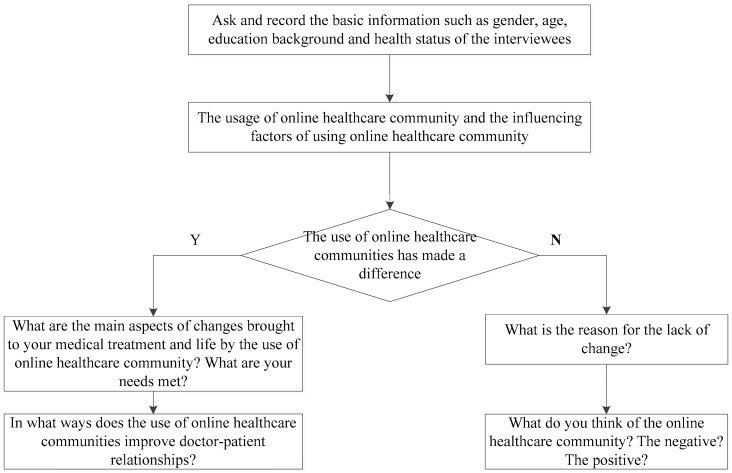
The online healthcare community uses an interview idea sketch.

Focusing on the core question of how the use of the online healthcare community affects the doctor–patient relationship, this paper first designed ideas for the interview and an outline for use. The specific steps were as follows. First, the relevant literature was examined, and discussions were held with two teachers and two doctoral students on the team, and the preliminary interview outline was designed on this basis. Second, a teacher on the team listed the important questions, drawing on the draft interview outline. This process was then repeated with several other groups of teachers and doctoral students until the lists of questions converged. The interview ideas that were adopted in this paper are given in Figure 2.

This interview outline includes the following two main parts. First, the purpose of the interview is explained to the interviewees, and then the interviewees are asked for their basic information, including gender, age, educational background, monthly income, type of employment, and health status. Second, the reasons cited by those who used online healthcare communities and their suggestions for improving the doctor–patient relationship through online healthcare communities were examined. The questions included the following: “What do you know about online healthcare communities?” “Have you ever used an online healthcare community?” “What are the main uses of online healthcare communities?” “What factors have influenced your use of online healthcare communities?” “Does the healthcare provided through the online healthcare community meet your needs as well as traditional healthcare care? What needs do they meet?” “How can the use of an online healthcare community improve doctor–patient relationships?” Table 1 lists the interview questions.

This article used snowball sampling to recruit interviewers. We mainly focused on those users who have used the online healthcare community. The specific process was as follows: first, selected classmates and friends and used them as seeds for the author’s city of Jinan, China. If these contacts had the experience of using the online healthcare community, we briefly described this study and asked them whether they would be willing to participate in the study. If the interviewers had no objection, everyone who agreed was tentatively selected as an interviewee, and an in-depth interview was conducted, following the interview outline designed in the early stage. The interviewers were requested to participate in the assistance truthfully and confidently. The interview content was limited to the current topic of research. The interviewees were required to assist in the research to present their true feelings and opinions. The private information of the interviewees involved in the interview was anonymized and desensitized. Finally, at the end of the interview, the interviewees were requested to recommend suitable personnel as prospective interviewees. Following the interview outline developed as described, 18 participants in Jinan, Liaocheng, Linyi, Heze, and other cities were selected for interview from October 2020 to April 2021. Table 2 lists the basic information for the interviewed personnel. The interviews were conducted face-to-face at first, and online interviews were used to supplement unclear or emerging issues. Each interviewee was interviewed for about 40 to 90 min. The entire interview process was recorded with the advance consent of the interviewee, and all the recordings were converted into documentary records.

## 3. Results

### 3.1. Open Coding

Open coding is used to analyze and organize the collected data to form a conceptualization and categorization [16]. For this, it is key that the researchers analyze the original materials and sort them out from the full interview recordings and create summative concepts. When certain concepts have the same or similar characteristics, these concepts are integrated. On this basis, it is refined into a higher-order abstract category (called “categorization”) [17]. In this paper, open coding and concept extraction are carried out for 2/3 samples. The partial open coding results are given in Table 2.

The interview data are sorted out sentence by sentence using open coding, and a total of 63 concepts are extracted. The concepts are combined according to the principle of relevance or similarity to form 53 categories, as shown in Table 3. To take the platform function design category as an example, this paper identifies the following preliminary concepts from the interviews: The platform function is not perfect; it is recommended to set a reminder function; there are not many reservation items on the platform; the consultation is not specified offline, no specific examination can be set; it is recommended to set a reply reminder window; as a supplement to a doctor’s visit, the platform needs to be improved. The interviewees asserted that the platform function needed to be improved. The paper summarizes this type of response into the category of platform function design.

### 3.2. Axial Coding

The purpose of axial coding is to analyze the different relationships between concepts and categories and discover and construct relationships [18,19]. Typical association paradigms involve causality, action/interaction, time sequence, context, type, structure, and so on. This paper analyzed the relationship among the 53 initial categories obtained from open coding, and 24 main categories can be integrated through reclassification based on multiple dimensions, such as patients, doctors, hospitals, online community platforms, and the healthcare environment, as shown in Table 4. 

Healthcare cost refers to the comprehensive expenditure of economy, time, and energy in the process of seeking healthcare consultation. It includes two main sub-categories: providing free consultation services online and reducing healthcare costs online. Healthcare distance refers to the distance between the patient’s geographical location and the nearest healthcare institution. The degree of disease risk refers to the severity and urgency of the disease when the patient seeks healthcare consultation, which mainly includes three sub-categories: disease category, disease symptoms, and disease urgency. The impact of major emergencies refers to the impact of public health, extreme weather, social security, the natural environment, and other emergencies on the way patients seek healthcare advice, such as the impact of COVID-19 on the way patients seek healthcare advice. Patient evaluation refers to the conclusions that a patient makes through a comprehensive judgment and analysis of the healthcare services provided by the doctor based on his or her own healthcare experience, which includes the three sub-categories evaluation effectiveness, evaluation authenticity, and evaluation credibility. The hospital grade is the conclusion of hospital qualification evaluation based on the hospital’s floor area, building area, outpatient area, fixed assets, clinical departments, the number of wards, number of beds, talents, technical force, and other indicators. 

Healthcare resources refer to the sum of various production factors, such as healthcare personnel, healthcare expenses, healthcare beds, healthcare facilities and equipment, knowledge and skills, and information that healthcare institutions can provide healthcare services. It mainly includes three sub-categories: unbalanced resource distribution, tight healthcare resources, a large gap between urban and rural healthcare resources, and more famous doctors on online healthcare platforms. Hospital facilities refer to the overall layout, color decoration, vehicle management, modern healthcare equipment, and management facilities of the hospital to meet the needs of healthcare workers to adapt the healthcare environment and healthcare functions. It mainly includes two main sub-categories: hospital structure layout and supporting facilities. Healthcare treatment refers to the steps and main procedures that patients need to go through in seeking healthcare treatment. It mainly includes two sub-categories: offline healthcare treatment process and online healthcare treatment process. 

Doctor–patient communication refers to the communication and exchanges between patients and their family members and healthcare staff in healthcare institutions on diagnosis and treatment, services, health, psychological, and social factors in the process of seeking healthcare advice. It includes six sub-categories: emotional attitude, communication mode, communication adequacy, communication efficiency, communication content similarity, and communication service quality. Platform convenience means that functions provided by online healthcare platforms can meet users’ needs more conveniently and quickly. The rich information resources of the platform mean that the online healthcare platform can provide a variety of information resources such as hospitals, doctors, appointment registration, consultation, Chinese and Western medicine, healthcare experience, and patient evaluation, especially when patients face difficult and complicated diseases, they can choose well-known expert doctors for consultation from the whole country. This mainly includes two sub-categories: informational richness and access to healthcare resources. 

Platform design refers to the design of platform functions and platforms functional interfaces by platform operators adhering to the patient-centered positioning concept to ensure that the functions provided by the platform are easy to use and practical, and the interface is warm and friendly, which mainly includes two sub-categories: platform function design and platform interface design. Platform visibility refers to the degree to which the online healthcare platform is known and understood by patients, and it is an objective measure for evaluating the reputation of the platform. Platform trust refers to patients’ positive expectations of online healthcare platforms and their belief that online healthcare platforms are reliable when they experience healthcare services through online healthcare platforms. It mainly includes four sub-categories: platform authority, platform reliability, platform background strength and healthcare knowledge popularization. 

Platform information security refers to a series of technical and management measures adopted by the platform to ensure that data will not be damaged or leaked due to accidental or malicious reasons. It mainly includes four sub-categories: system vulnerability, information disclosure, privacy information protection, and hidden disease information protection. Doctor trust refers to patients’ positive expectation of doctors and their belief that doctors are reliable in the process of experiencing healthcare services. It mainly includes three sub-categories: information disclosure, qualification level, and diagnosis and treatment experience. The professional quality of doctors refers to the comprehensive quality that doctors show in their professional work after systematic vocational training. It includes two sub-categories doctors’ attitudes and healthcare ethics. Doctors’ skill refers to the skills and the ability to use technology that doctors have mastered following systematic vocational training. 

Healthcare regulations and policies are the policies and normative documents formulated to promote the healthy, stable, and orderly development of the healthcare industry. It includes the two sub-categories of policy and legal support and healthcare system guarantee. Patient satisfaction refers to the patient’s expectation of healthcare care services due to the requirements of disease, physical health, and other aspects, as well as the evaluation of healthcare care services based on the healthcare experience. Healthcare quality perception refers to the quality and effect of healthcare services, mainly refers to the timeliness, effectiveness, and safety of healthcare services. Doctor–patient relationship mainly refers to the relationship between patients and doctors formed in the process of healthcare service. Emotional dependence refers to the results-based emotional evaluation generated by patients in the process of healthcare service interaction, which reflects the emotional connection and bond established between patients, doctors, and online healthcare platforms. It includes two sub-categories of emotional support and psychological comfort.

### 3.3. Selective Coding

The main task of selective coding is to refine the core category through systematic analysis in the identified category, find suitable storylines to establish the correlation between the core category and other categories, and finally build the theory [20,21]. An analysis of the interview data showed that the interviewees mentioned doctor–patient communication, healthcare process, and healthcare cost most often. Therefore, doctor–patient communication, healthcare process, and healthcare cost were taken as the core categories. The use of the online healthcare community can be analyzed from the perspectives of patients, doctors, hospitals, platforms, and the healthcare environment. 

The patient perspective includes three major dimensions: degree of disease risk, patient evaluation, and healthcare distance. The disease risk degree includes three dimensions: disease category, disease symptoms, and degree of emergency, and the patient evaluation include the three dimensions of evaluation utility, evaluation authenticity, and evaluation credibility. The doctor’s perspective includes three dimensions: the doctor’s trust, the doctor’s professional accomplishment, and the doctor’s skill. A doctor’s trust includes the three dimensions information disclosure, doctor’s qualification level, and diagnosis and treatment experience. A doctor’s professional accomplishment includes two dimensions: the doctor’s attitude and the doctor’s ethics. Platform perspective includes convenience, platform, information resources, platform design, platform awareness, trust and six big dimensions of the platform, information security platform, information resources include information richness and healthcare resources acquired two dimensions, platform design, including function design, and interface design of two dimension platform. Platform visibility includes the two dimensions platform publicity and platform promotion, platform trust includes four dimensions of platform reliability, platform authority, platform background strength and popularization of healthcare knowledge, and platform information security includes the four dimensions information disclosure, system vulnerability, privacy information protection, and hidden disease information protection. 

The hospital perspective includes three dimensions: hospital level, medical resources, and hospital facilities. Among them, healthcare resources including healthcare resource distribution is not balanced, healthcare resources nervous, a large gap between urban and rural health resources, and online platform leading doctors numbers; the structure and layout of hospital facilities, including hospitals and hospital facilities dimensions; the perspective of the healthcare environment includes the impact of major emergencies and guarantee of the healthcare regulations and policies, which include policy and legal support and the healthcare system guarantee. Doctor–patient communication includes the seven dimensions emotional attitude, communication mode, communication adequacy, communication style, communication efficiency, communication content similarity, and communication service quality. The Healthcare treatment process includes two dimensions: offline healthcare treatment process and online healthcare treatment process. Healthcare cost includes two dimensions: providing free consultation services online and reducing healthcare costs online. Emotional dependence includes two dimensions: emotional support and psychological comfort.

**Figure 3:**
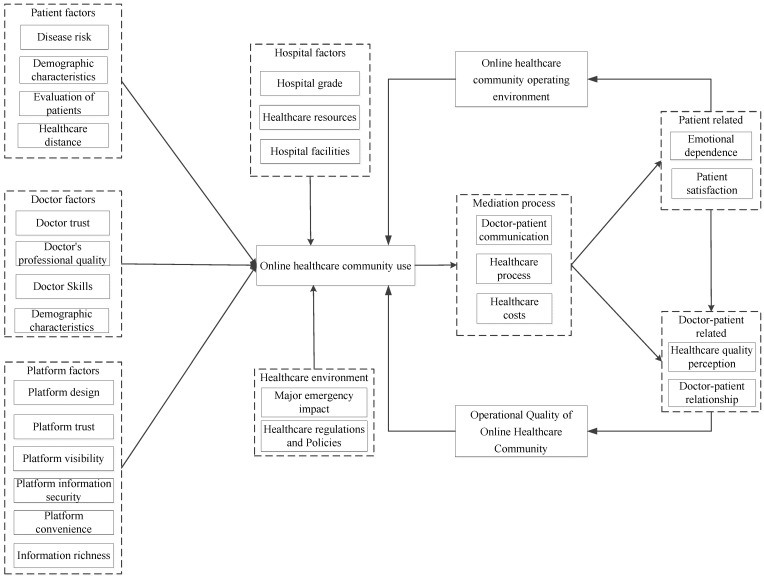
Antecedents and consequences of online healthcare community usage.

Figure 3 shows the “storyline” of the impact of the use of online healthcare community on doctor–patient relationship: Patients using online healthcare community by doctors, hospitals, and the healthcare community online platform itself and the impact of the healthcare environment, when patients after using the online healthcare community, to enhance its strength of the doctor–patient communication, healthcare treatment process optimization, healthcare costs are lower, thus improve patients experience, namely the emotional attachment, and improve patients satisfaction. When patients’ healthcare experience is improved, the perception of healthcare quality is ultimately improved, and the doctor–patient relationship is improved. At the same time, interview data also showed that the improvement of doctor–patient communication, the optimization of the healthcare treatment process and the reduction of healthcare costs after patients use the online healthcare community can also improve the perception of healthcare quality and improve the doctor–patient relationship. Finally, the improvement of patient’s healthcare experience and doctor–patient relationship will produce feedback on the operation quality and operating environment of the online healthcare community, promoting the promotion and use of the online healthcare community.

## 4. Theoretical Saturation Test

Previous studies have found out that indexes to evaluate the level and quality of case studies include intrinsic validity, extrinsic validity, and reliability [22]. To test the rationality and validity of case studies, this study adopts various methods. 

To improve the level of reliability and validity, when the case reaches theoretical saturation, it is used as the criterion to stop the analysis. Theoretical saturation means that the theoretical construction tends to be saturated when the subsequent interview data cannot further generate new concepts or obtain new categories [23,24]. In this paper, into the interview results, 2/3 of the total samples were randomly selected for detailed coding analysis and model construction, and the remaining 1/3 samples were used for a theoretical saturation test. The study shows that no new concepts and categories and no new relationships among categories were seen when the remaining 1/3 samples are used for the theoretical saturation test. Therefore, the theoretical construction of this paper has reached saturation.

## 5. Conclusions

From a theoretical point of view, this paper is an exploratory study of the use of online healthcare communities and its impact. The antecedents and consequences of online healthcare community use are still unclear. The existing results are not systematic, and comprehensive enough to study the use of online healthcare communities and their impacts. Unlike existing studies, this paper collects first-hand data on the use of online healthcare communities through in-depth interviews and gradually extracts the key factors that affect the use of online healthcare communities. What’s more, this paper also reveals the mechanism of action of online healthcare community use. The conclusions of this paper have important theoretical and practical significance for the development of online healthcare community platforms and the sustainable development of doctor–patient relationships.

This paper includes certain limitations and indications for future directions. The details are as follows: first, due to limitations of time and ability, only 18 respondents were interviewed. Although these samples are sufficient in theory, future research can increase the number of samples to further expand the identification of antecedents and consequences of online healthcare community use. Second, in the encoding of grounded theory, due to limitations in ability, there may be different grounded results. Future research may make use of quantitative research methods, such as questionnaires, to verify the research conclusions of this paper. Finally, this paper only provides a preliminary exploration of the antecedents and consequences of the use of online healthcare communities in qualitative research. From the research framework of this paper, future research should be able to further explore the influencing factors and processes of the use of online medical communities, further exploring the impact of the mechanism of use of online medical communities.

## Figures and Tables

**Table 1:** Interview question list.

Items	Main Interview Questions
1	Please briefly introduce your personal information, including gender, age, education background, monthly income, nature of employer, and health status.
2	How much do you know about online healthcare communities, including categories, advantages and disadvantages, and what problems can be solved?
3	Have you used the online healthcare community? Why do you want to use it? What is the main purpose of using the online healthcare community? Why not?
4	What factors influence your use of online healthcare communities?
5	What changes will the use of online healthcare community bring to your healthcare treatment and life?
6	Compared with traditional healthcare services, can the healthcare services provided through the online healthcare community meet your needs? What needs are met?
7	What do you think about using online healthcare communities? Positive/negative aspects.
8	What do you think are the main factors affecting the doctor–patient relationship? In what ways can the use of online healthcare communities improve doctor–patient relationships?

**Table 2:** Partially open coding results.

Original Statement	Preliminary Extraction of Concepts
…It turned out that my mother was ill and was treated in Beijing, and then I used Jingyitong……I think it was very convenient to sign up, and it is more convenient to make an appointment…	Easy to make an appointment
…Through this, I can compare the symptoms to understand which doctor may be more suitable for my disease or my symptoms, and then there are some other patients’ comments on the doctor below him…	Choose a more suitable doctor and view patient reviews.
…Because I feel that Jingyitong does a better job, that is, most hospitals in Beijing have doctors on it, it is a collective…	Many well-known doctors
…Then some are like some big hospitals in Beijing, the structure layout and process are more complicated. In that building, let alone the elderly, even young people, like me, may not be able to figure it out when they go to the hospital for the first time…Because when I took my mother to see a doctor at Tiantan Hospital for the first time, I couldn’t find the elevator in the International Department. You sometimes ask the service staff of the hospital. Although he provides good service, he can’t explain everything, perhaps the hospital is very large, and the patient can’t remember the route he was told…	Whether the offline hospital layout is reasonable and whether the process is reasonable; although the attitude of healthcare staff is good, they still cannot meet the needs of patients, resulting in dissatisfaction.
…But like last year’s epidemic situation, we can no longer go to the hospital to get medicine…Then I used the online healthcare community platform, uploaded her ID card, and uploaded her case, which is equivalent to having the medicine delivered to her home after diagnosing her online…	Take medicine online during the epidemic.
…So, I feel that there are some common diseases, some basic diseases, the kind that can be done online without doing some examinations or mainly to relieve some emotions, anxiety arise, but I think that this should be fine.	Disease category
…It is difficult for people from many cities in Shandong to come to Jinan to see a doctor, but there are some diseases that cannot be cured in other places…The online healthcare community may be more suitable for them, because compared to Jinan in Shandong, this healthcare resource is relatively good…	Online healthcare community is more suitable for remote areas, healthcare distance and geographical location
…That is to say, the biggest flaw of this platform may all have this flaw. The doctor may not know much about your physical and mental feelings, because you can only say something objectively or quantitatively, and he cannot understand your feelings. Because it has only one contact or communication in a written language…	Online communication involves less emotional contact
…For example, he said that you injected insulin, say 1 mg, and then the blood sugar measured later was not that high. He then said that you injected 0.5 mg, and then the blood sugar dropped after the injection, and then it may appear. I was diagnosed with hypoglycemia, but the doctor said that you need to inject 0.1 mg. He didn’t ask any reason and immediately lowered the data, so I thought this answer was sloppy at the time… …I think that some of his students or other people are replying to him, because at the time he said he did not see that much information, he may have assistants or the kind of graduate students he brought… …I am not very relieved, and I think they are all pre-set computer programs. He searches for keywords, such as your blood pressure and your blood lipids, and he will give you that kind of mechanical reply…	The doctor’s response is sloppy Feeling that it is not the doctor himself who is replying online Suspicion that it is a program set in advance, mechanical reply
…I chose it myself, because when we went to the offline hospital to see a doctor, we hung up on his number, and after we came back, we learned about the consultation through him on the internet. From beginning to end, we passed through his hand, which is relatively familiar…	I saw the doctor offline and continue to consult online in the follow-up
…He also advised me at the time that if you can go to the hospital to find the doctor, don’t go online, because what he told me is that there may be some teams behind the scenes in the online healthcare community that are helping him manipulate some things, such as answering questions, although his may be the official answer…	There may be team operations and distrust of the platform
…I think if the functions of these platforms are improved, for example, such as through the creation of reminders, after you register, make an appointment to queue up for B-ultrasound examination…There’s a line in front of him, and he doesn’t have that kind of reminder, does he? It wants to remind you that it’s almost here, hurry up and wait for these functions. I think he is not fully functional yet, and it is also limited to some inspections, not all healthcare examinations can be carried out online…	The platform function is not perfect; it is recommended to set a reminder function; there are not many reservation items on the platform
…There is another point. During the epidemic, I was not pregnant. There were some obstetric examinations, but there were some symptoms in the later period. You can’t go to the hospital all the time. When the epidemic was the worst, you were encouraged to use the online healthcare community. Once, I couldn’t go to the hospital because of the epidemic, and the symptoms were not particularly obvious, but I was anxious to consult. I think it can also alleviate healthcare anxiety…	Because the epidemic is inconvenient to go to the hospital, the symptoms are not obvious (not serious), and I am more anxious
…I only contact doctors I am familiar with in the online healthcare community, like when I went to the Provincial Women’s and Children’s Hospital, he asked me to scan the code to recommend it, and then I would contact him. This is because of trust, so I chose them ask some questions…	Need to trust the doctor
…I was wondering if this could be the case... You said that he accumulated experience or whatever, he has time, and then he can also use the platform to allow him grow his knowledge and increase his healthcare experience…	Accumulate healthcare experience
…To be honest, I think that the reason that I would not use online healthcare care under the same conditions is that registering information will indeed leak information, I think it will, so I don’t want to use it. Another is that I think my physical condition is relatively sensitive information, and I don’t like to use this method online, and I don’t think it is very safe…	Worry about information leakage
…You are like this online healthcare community; I don’t think the information given by the hospital is as accurate as the information given by the hospital. For example, some doctors, whose information is not as authoritative and scientific as the information given by the hospital, may he the attending doctor or the chief doctor. What kind of scientific research has been used to treat what diseases? I think that sometimes the information update of this online healthcare community may not be as authoritative and quick as the hospital…	Information is inaccurate; information updates are not regular, not as authoritative as physical hospitals
…When you don’t understand so deeply and understand so much, you don’t naturally have that kind of natural trust. It’s not like going to a hospital, which is certified by the state. If you are like a district hospital, it is not as good as a provincial hospital, so there are quite a few platforms such as online healthcare communities, but I don’t know which one is the best? Then you don’t know how to choose. To be honest, there may be another one that you don’t know about, so there is no such natural trust.	Trust in the community; there are many online healthcare communities, I don’t know how to choose
…I think that it is this kind of online. It must be that your needs are very clear. You will not ask him what is going on. I just want cephalosporins, I just want this, and then I will take it, so I say this is more convenient…	If the patient’s needs are clear, the online platform is efficient
…I want to use the online healthcare community because I think it may supplement, for example, my visits to the hospital to communicate with the doctor…	A supplement to offline communication
…Especially if you have been queuing offline for a whole morning, it may only take a few minutes for you to wait in line. At that time, when you were nervous or in a hurry, you didn’t ask a lot of questions. After you go back, you can use the online healthcare platform to ask them. So sometimes I think it’s good, and sometimes I can get a more satisfactory opinion. This is communicated better…	The offline consultation time is short and the communication is insufficient; there is better communication online
…You see, when I went to the provincial hospital, the doctor recommended two platforms. One of them was Good Doctor Online. If you have some knowledge about this platform, such as its popularity and publicity in various aspects, you may have some understanding, and then we prioritized Good Doctor Online…	Platform publicity and popularity
…There are generally two situations in which an online platform is used. In the first, you have never gone online to see a doctor. It may be that the specific situation requires a hospital visit, and some doctors may say that you should go to the hospital that is closer to you. This is the first case that you have never seen an offline doctor. The second situation is that I have seen what I just said, and he may also understand your condition. I have chosen a doctor who will treat you offline; so, he will be able to be more detailed, as he may be aware of all aspects of the patient’s information…	There has never been offline consultation and consultation, and the doctor’s words are relatively shallow or relatively broad; As an offline supplement, it is more detailed
…Because the doctor who replied to us was surnamed Kong, and Dr. Kong had already told us that during the day he was an outpatient clinic for one day, and then he calmed down to reply to us at 9 o’clock in the evening, and he also apologized to us. I think he should be during the day. very busy, attitude is good…	Online doctors have a good attitude
…A good doctor online will only give three free meetings, and then he will start to charge…	Free consultations are limited in number
…There is a special hurry. If I am not in a hurry, I can take my time and spend my energy offline. The reason that I go online is because I want to get a particularly urgent answer or be particularly anxious…	If you are in a hurry or don’t have time to go to the hospital, use the online healthcare community
…On the online healthcare platform, a lot of good doctors are mentioned online, and sometimes they search for some symptoms, and he will come up with a lot of this. I think that kind of doctor I don’t really trust his qualifications or his level…	Doubt of the qualifications and skill level of online doctors
…The most important thing is that I live in Changqing now. You go to the provincial hospital. Sometimes it takes a day for you to do an examination, because some things have been checked, you may not be able to pick them up until 24 h later, so your entire cost is very large. Online appointments can reduce healthcare costs in terms of time and economy…	Reduce healthcare costs (time, effort, and economics)
…Can the degree of communication between doctors and patients be increased? The online healthcare community makes this possible, and the satisfaction of patients can be increased. As for the doctors we have had experience with, I think they are all quite satisfied. They can improve the level of communication, improve the patients’ satisfaction, and basically meet our expectations through our consultation…	Improve doctor–patient communication and improve patient satisfaction
…The quality of healthcare care and healthcare effect, anyway, these questions I asked must be answered, and if they are answered, the healthcare effect will be achieved…	The quality of healthcare care has also improved
…You see, when I was pregnant at the time, I found a doctor to examine me specially, and then one day I suddenly had a problem, I wanted to go to him, he might not be there, right? Using the online healthcare community, I can find him at any time, and I trust him very much. Through these two online communications, he responded to me in time, and I trust him even more. I think this has altered, and the doctor–patient relationship is also very good. I am more confident in a certain doctor, and I think it is better anyway…	Increased trust Improved doctor–patient relationship
…Trusting the doctor may even mean more trust in their entire hospital, other doctors in the hospital, and some of the healthcare services the hospital provides. There is some trust in this, right? For example, if you go to the Municipal Women’s and Children’s Hospital, after having the experience of using the online healthcare community, you may say that you will trust the hospital more, right?	Improved doctor–patient relationship
…If the disclosure of personal information is excluded, I think it is still ok. That is to say, at least you have one more way, although it is not the mainstream, but at least it can be used as a supplementary way. I think that if the platform has done a good job, or if the country has certified such a few platforms, I will trust it very much…	Worry about personal information leakage; As a supplement to healthcare treatment; The platform needs to be improved
…Some doctors may exhibit a good attitude, be very kind, and have a high level of skill; they may communicate with you in a caring way, and you may develop a strong emotional bond or emotional dependence on this type of doctor…	Psychological comfort
…The doctor at the provincial hospital for women and children said that you scanned my code because of the state of the pandemic, but if you think that question is not particularly urgent but you need to know, you can send me a message…	Emotional support
…Later, I learned that there is such a channel; I think this channel is okay, because although it does cost money, it also opens a channel for communication with well-known doctors, and there is no other way, although it will increase healthcare treatment itself. It does have a cost, but this may vary depending on whether it is an intractable disease or a major disease, whether money is a major problem for the patient, or whether the patient can find a doctor with sufficient healthcare skills, maybe this is the main problem…	Especially in the face of difficult and miscellaneous diseases, the cost is secondary, and the ability to choose well-known experts is the main issue
…I feel that the service is still relatively complete, and people are approaching it thoughtfully, so that one of us finds it to be convenient, and another may also feel that it is very warm, the whole interface he made, including some tweets. Language is from the perspective of caring and caring for patients, so it feels good…	The online service is complete, the interface is warm and friendly, and the patient’s emotions are maintained
…For example, in the process of seeing a doctor, the face-to-face communication between the doctor and you are different from your online communication. First, you must pay attention to hearing, listening, and inquiring, right? If you say that there are some very small differences in your state, it does not mean that you can judge this through access to the online doctor, and then the communication process will also affect some aspects of you. Even with video, there may be a gap between offline and face-to-face…	Dissatisfaction of the online healthcare community: the consultation is not specific offline, and the specific examination cannot be done
…I don’t know if he has any push here, like advertising or something…	Worry about advertising push
…Another good thing about the online healthcare community is that after the Jingyitong that I follow, it sometimes has popular science knowledge that is good for study. Anyway, some popular science knowledge, such as how to eat and cure this season, or what are the common high diseases, sometimes the doctor will give me some of this, but sometimes I think that it is pretty good. It has some healthcare knowledge, it is not that it is very obscure, it is general truth, it is simple and concise…	Popularization of science knowledge Increase the favorability of the platform
…There may be system loopholes on the platform, but it is unlikely to be leaked maliciously, and the platform also needs to maintain its own brand. There is a certain risk due to technical loopholes…	System vulnerabilities and hidden dangers
…I doubt whether they will sell data, because the platform needs to make a profit, but they still think that the healthcare resources should be mainly for public welfare, so, after mixing the profitable part, you will naturally be right. He felt a sense of resistance. Maybe people do not really sell your data or make money in this area, but you have this suspicion…	Where is the profit for the platform? Concern about data security
…Is the online healthcare community not very good for healthcare reimbursement, such as registration fees and other expenses? I don’t know if it is, if we are in the hospital, he can reimburse…	Online healthcare expense reimbursement
…From the point of view of the present, the responses are a little late, and there may be time deviations. For example, I waited for his reply during the day, but he did not reply. go and see. Because it’s not like WeChat, it has prompts that you must open to see. Maybe I didn’t watch it for several days because I forgot, so it didn’t prompt you that it responded. I think there could also be a popup in the background of the platform…	It is recommended to set a reply reminder window
…I often read patient evaluations, and sometimes I also think about evaluations, whether some of them are true or not, and some are just like Taobao’s review of positive reviews or something. I also hope that if the background can review some cases of this patient, or indeed prove the basic situation that he has seen a doctor in this place, the evaluation may be more credible…	Whether the patient’s assessment is credible, it is recommended that the platform audit be more stringent

**Table 3:** Results of category extraction.

Category	Concept
Free online consultation service	The number of free consultations on online healthcare platforms is limited
Online healthcare treatment costs can be reduced	Reduce healthcare costs (time, effort, and economics)
Healthcare distance	Online healthcare community is more suitable for remote areas, healthcare distance and geographical location
Disease category	Disease category
Disease symptoms	Because the epidemic is inconvenient to go to the hospital, the symptoms are not obvious (not serious), and more anxious
Emergency level	If you are in a hurry or don’t have time to go to the hospital, use the online healthcare community
The impact of the epidemic (the impact of major public health events)	Take medicine online during the epidemic
Evaluation of the utility	Patient evaluation is important in choosing a doctor
The authenticity of the review content	True and credible of patient evaluations
Evaluation credibility	Is the patient evaluation credible?
Hospital size and grade	Hospital size and grade
The online platform has many well-known doctors	Many well-known doctors
Uneven distribution of healthcare resources	Healthcare resources are tight and unevenly distributed
Healthcare resources are tight	Hospital beds are tight (healthcare resources are tight)
Large gap between urban and rural healthcare resources	Regional healthcare resources and healthcare level gaps are too large (unbalanced)
The hospital layout	Is the offline hospital layout reasonable? Although the attitude of healthcare staff is good, they still cannot meet the needs of patients, resulting in dissatisfaction.
Hospital supporting facilities	Hospital supporting facilities and convenience guide map
The offline healthcare treatment process is cumbersome	Healthcare treatment process (do not interrupt the healthcare treatment process)
Online platform optimizes healthcare treatment process	Online registration, appointment inspection items (shortening the time for healthcare treatment), and optimizing the healthcare treatment process
Emotional attitude	Online communication has less emotional contact
Way of communication	A supplement to offline communication
	Offline treatment, follow-up online consultation
Online communication adequacy	The offline consultation time is short, and the communication is insufficient; better communication online
Communication efficiency	Online platforms are efficient when the requirements are clear
Communication content similarity	Browse doctor–patient communication records
Communication service quality	Never have offline consultation, the doctor said relatively shallow; as an offline supplement, more detailed
Convenience	It is convenient to make an appointment and register, and it is easy to view and browse
Informative	Provision of information on a doctor, selection of a more suitable doctor, and can also view patient evaluation, understand the doctor convenient registration
Access to healthcare resources	Particularly in the face of difficult and little-known diseases, the cost is secondary, and the ability to choose well-known experts is the main
Platform function design	The platform function is not perfect; it is recommended to set a reminder function; there are not many reservation items on the platform
	Dissatisfaction of the online healthcare community: the consultation is not specific offline, and the specific examination cannot be done
	It is recommended to set a reply reminder window
	As a supplement to a doctor’s visit, the platform needs to be improved
Platform interface design	The online service is complete, the interface is warm and friendly, and the patient’s emotions are maintained
	Platform interface design; whether to design from the perspective of patients
Platform publicity	Platform publicity and popularity
Platform promotion	Hope that the hospital will actively connect with the platform and promote doctors
Platform reliability	There may be team operations providing responses and concomitant distrust of the platform: if the doctor’s response is sloppy; Feeling that a non-doctor is responding; Suspect it is a program set in advance, mechanical reply
	Concern about advertising push
	To determine whether the patient evaluation is credible, it is recommended that the platform review be more stringent
Platform authority	The information is inaccurate; the information is not updated in a timely manner; and it is not as authoritative as the physical hospital
Platform background strength	Trust in the community; there are many online healthcare communities, and it is difficult to know how to choose
Popularization of healthcare knowledge	Popularization of science knowledge Increase the favorability of the platform
Information leakage	Concern about information leakage
	Concern about personal information leakage
	Where is the profit point of the platform? Concern about data security
System vulnerability	System vulnerabilities and hidden dangers
Privacy protection	Permission settings, privacy protection; Strengthening privacy protection (desensitization of key information)
Hidden disease information protection	Disease privacy information protection
Information disclosure	Easy to view and browse
Doctor’s qualification and level	Doubt the qualifications and level of online doctors
Diagnosis and treatment experience	Accumulated healthcare experience
Doctor’s attitude	Online doctors have a better attitude
	Doctor’s attitude
Healthcare ethics	Healthcare ethics
Doctor skills	Doctor’ skills (healthcare level)
Policy legal support	Need policy, ethics, legal support
Healthcare system guarantee	Online healthcare expense reimbursement
Patient satisfaction	Improve doctor–patient communication and improve patient satisfaction
Perception of healthcare quality	The quality of healthcare care has also improved
Doctor–patient relationship	Improved doctor–patient relationship
emotional support	Emotional support
psychological comfort	Doctor care, psychological comfort

**Table 4:** Main category and category connotation formed by the main axis coding.

Main Category	Subcategory	Connotation
Healthcare costs	Free online consultation service	Online healthcare platform provides free consultation service
	Online healthcare treatment costs can be reduced	Online healthcare platform can save time and reduce healthcare cost (time, energy, and economy)
Healthcare distance	Healthcare distance	The distance between the patient’s geographic location and the nearest healthcare facility
Disease risk	Disease category	Disease category
	Disease symptoms	The symptoms of the disease are not obvious (not serious) but provoke anxiety
	Emergency level	Anxious to seek healthcare advice after illness
Major emergency impact	Epidemic impact	The impact of COVID-19 on access to health care
Patient evaluation	Evaluate utility	The impact of patient evaluation on other patients’ choice of physician
	Evaluate authenticity	Whether the content of the patient’s evaluation is true
	Evaluation credibility	Is the patient evaluation credible?
Hospital grade	Hospital size and grade	Conclusions on the evaluation of hospital qualifications based on hospital scale
Healthcare resources	Uneven distribution of healthcare resources	Unbalanced geographical distribution of healthcare resources
	Healthcare resources are tight	Healthcare resources such as healthcare equipment, diagnosing and treating doctors, and inpatient beds are in short supply
	Large gap between urban and rural healthcare resources	Regional healthcare resources and healthcare level gaps are too large (unbalanced)
	The online platform has many well-known doctors	The number of well-known doctors in the online healthcare platform is large
Hospital facility	Hospital structure layout	Offline hospital structure layout, department arrangement is scientific and reasonable
	Hospital supporting facilities	The hospital has perfect supporting facilities and clear signs for the convenience of the people
Healthcare process	Offline healthcare treatment process	There are many offline healthcare consultation links, the healthcare treatment process is cumbersome, and the healthcare treatment process is easily interrupted
	Online healthcare treatment process	Online registration, appointment inspection items and other functions provided by the online platform can shorten the time for healthcare treatment and optimize the healthcare treatment process
Doctor–patient communication	Emotional attitude	Emotional contact in the process of doctor–patient communication
	Means of communication	After treatment in offline physical hospitals, online consultation is to be continued as a supplement to offline communication
	Communication adequacy	The offline consultation time is short, and the communication is insufficient; better communication online
	Communication efficiency	Online communication, timely response, and high communication efficiency
	Communication content similarity	Seek help by browsing other doctor–patient communication records
	Communication service quality	As a supplement to offline communication, communications are more detailed
Platform convenience	Convenience	Online appointment and online consultations are convenient and fast; patients can also inquire online and register offline for treatment
Rich platform information resources	Informative	Information such as doctor information, registration information, consultation information, drug information, healthcare treatment experience, and patient evaluation can be obtained
	Access to healthcare resources	When faced with intractable diseases, you can choose well-known expert doctors
Platform design	Platform function design	Designed from the perspective of patients, the platform functions are more perfect
	Platform interface design	The online service of the platform is complete, and the interface is warm and friendly
Platform popularity	Platform publicity and promotion	The platform carries out external publicity through various means to enhance the promotion efforts of the platform
Platform trust	Platform reliability	Patients’ trust in online healthcare platforms
	Platform authority	Patients have a sense of trust in the platform and will not question the platform
	Platform background strength	Recording and certification qualifications obtained by platform operators
	Popularization of healthcare knowledge	Popularization of popular science knowledge, data update of unexpected public security incidents, and increased favorability of the platform
Platform information security	Information leakage	Patient personal information leaked
	System vulnerability	Platform system loopholes, there are hidden risks
	Privacy information protection	Permission settings to strengthen the protection of privacy information
	Hidden disease information protection	Desensitization of key information to strengthen the protection of hidden disease information
Doctor trust	Information disclosure	Doctors take the initiative to provide personal information to the platform, and allow the platform to use their personal information in an agreed manner
	Doctor’s qualifications and level	Healthcare professional and technical personnel obtain the qualifications for corresponding technical positions in accordance with relevant national and provincial regulations and conditions
	Diagnosis and treatment experience	Knowledge or skills accumulated in the course of many times of healthcare practice
Doctor’s professional quality	Doctor’s attitude	Psychological and behavioral tendencies of doctors towards patient groups
	Healthcare ethics	The professional ethics of healthcare staff
Doctor skills	doctor skills	Physician skills and ability to use technology
Healthcare regulations policy	Policy legal support	Policy support necessary for the operation and development of online healthcare platforms
	Healthcare system guarantee	Healthcare insurance system required for online healthcare consultation
Patient satisfaction	Patient satisfaction	Ratings of health care services based on experiences at visits
Perception of healthcare quality	Perception of healthcare quality	Quality of work in health care
Doctor–patient relationship	Doctor–patient relationship	The relationship between doctors and patients in the process of healthcare services
Emotional dependence	Emotional support	Being emotionally understanding and supportive
	Psychological comfort	Assessing the psychological and spiritual needs of patients, and try to meet them

## Data Availability

The data presented are included in this study; additional data may be provided by the corresponding author on request.
